# Handwriting Declines With Human Aging: A Machine Learning Study

**DOI:** 10.3389/fnagi.2022.889930

**Published:** 2022-05-06

**Authors:** Francesco Asci, Simone Scardapane, Alessandro Zampogna, Valentina D’Onofrio, Lucia Testa, Martina Patera, Marco Falletti, Luca Marsili, Antonio Suppa

**Affiliations:** ^1^IRCCS Neuromed Institute, Pozzilli, Italy; ^2^Department of Information, Electronic and Communication Engineering (DIET), Sapienza University of Rome, Rome, Italy; ^3^Department of Human Neurosciences, Sapienza University of Rome, Rome, Italy; ^4^Department of Informatic, Automatic and Gestional Engineering (DIAG), Sapienza University of Rome, Rome, Italy; ^5^Department of Neurology, Gardner Family Center for Parkinson’s Disease and Movement Disorders, University of Cincinnati, Cincinnati, OH, United States

**Keywords:** handwriting, aging, machine learning, convolutional neural network, telemedicine, smartphone

## Abstract

**Background:**

Handwriting is an acquired complex cognitive and motor skill resulting from the activation of a widespread brain network. Handwriting therefore may provide biologically relevant information on health status. Also, handwriting can be collected easily in an ecological scenario, through safe, cheap, and largely available tools. Hence, objective handwriting analysis through artificial intelligence would represent an innovative strategy for telemedicine purposes in healthy subjects and people affected by neurological disorders.

**Materials and Methods:**

One-hundred and fifty-six healthy subjects (61 males; 49.6 ± 20.4 years) were enrolled and divided according to age into three subgroups: Younger adults (YA), middle-aged adults (MA), and older adults (OA). Participants performed an ecological handwriting task that was digitalized through smartphones. Data underwent the DBNet algorithm for measuring and comparing the average stroke sizes in the three groups. A convolutional neural network (CNN) was also used to classify handwriting samples. Lastly, receiver operating characteristic (ROC) curves and sensitivity, specificity, positive, negative predictive values (PPV, NPV), accuracy and area under the curve (AUC) were calculated to report the performance of the algorithm.

**Results:**

Stroke sizes were significantly smaller in OA than in MA and YA. The CNN classifier objectively discriminated YA vs. OA (sensitivity = 82%, specificity = 80%, PPV = 78%, NPV = 79%, accuracy = 77%, and AUC = 0.84), MA vs. OA (sensitivity = 84%, specificity = 56%, PPV = 78%, NPV = 73%, accuracy = 74%, and AUC = 0.7), and YA vs. MA (sensitivity = 75%, specificity = 82%, PPV = 79%, NPV = 83%, accuracy = 79%, and AUC = 0.83).

**Discussion:**

Handwriting progressively declines with human aging. The effect of physiological aging on handwriting abilities can be detected remotely and objectively by using machine learning algorithms.

## Introduction

Neurodegenerative disorders, including Parkinson’s disease (PD) and Alzheimer’s disease (AD), represent a relevant global health issue, and the number of cases is going to increase steeply in prevalence in the next few decades (Armstrong and Okun, 2020; Feigin et al., 2020). Currently, the lockdown restrictions due to the COVID-19 global pandemic have challenged the clinical management of patients affected by neurodegenerative diseases, thus requiring innovative telemedicine approaches (Valdovinos et al., 2022). To this aim, it would be relevant to identify novel telemedicine tools allowing early and objective diagnosis, tracking the severity of the disease and thus improving the overall remote clinical management of patients with neurodegenerative diseases (Chirra et al., 2019). Practically, an ideal telemedicine setting should imply a safe, costly affordable and largely available tool able to easily collect biologically relevant information, in an ecological scenario.

As a possible innovative telemedicine strategy, in this study, we propose the remote assessment of handwriting. Handwriting indeed only requires safe, cheap, and largely available tools and can be simply collected in an ecological scenario. Moreover, handwriting samples can be easily digitalized by using smartphone-based high-resolution cameras and transmitted directly to a central hub for subsequent analysis. From a biological perspective, handwriting represents an acquired complex cognitive and motor skill resulting from the activation of a widespread brain network (Menon and Desmond, 2001; Lubrano et al., 2004; Purcell et al., 2011; Planton et al., 2013; Baldo et al., 2018; Chen et al., 2019; Bartoň et al., 2020). Indeed, in the clinical setting, handwriting is yet largely used to assess motor abilities in patients with PD (i.e., evaluation of micrographia and slowness of hand movements) and it is typically included in standardized clinical scales designed to examine cognitive functions in patients with dementia such as AD (Folstein et al., 1975). However, the potential application of handwriting analysis as a new telemedicine tool for neurodegenerative disorders crucially requires the preliminary assessment of a large dataset of healthy controls and the investigation of the effect on the handwriting of relevant biological factors such as physiologic aging.

Seminal studies using perceptual as well as objective kinematic analysis (Hilton, 1977; Dixon et al., 1993; Slavin et al., 1996; Walton, 1997; Contreras-Vidal et al., 2002; Teulings et al., 2002; Rodríguez-Aranda, 2003; Rosenblum and Werner, 2006; Burger and McCluskey, 2011; Caligiuri et al., 2014), have demonstrated age-related changes in handwriting skills in healthy subjects. However, to analyze a large amount of data for telemedicine purposes, automatically and objectively, more robust methodological approaches based on artificial intelligence are required for the classification of variables obtained from large datasets (Deo, 2015). For this purpose, machine learning has already been demonstrated to be a reliable tool for the assessment of handwriting in healthy subjects (Pang and Yang, 2016; Guo et al., 2021; Pei and Ouyang, 2021). Moreover, handwriting analysis based on machine learning has allowed inferring several features, including gender (Illouz et al., 2018), left/right-handedness (Al-Maadeed et al., 2013), presence of dysgraphia (Asselborn et al., 2020), specific personality traits (Lemos et al., 2018), and finally individualized fingerprints (Srihari et al., 2001). Also, preliminary studies using handwriting analysis with machine learning have demonstrated the possibility to predict age in healthy subjects (Zouaoui et al., 2017; Basavaraja et al., 2019).

We here investigated possible age-related changes in handwriting, objectively, in a large cohort of healthy subjects. To this aim, we examined and compared a simple handwriting task, collected in a real-life setting in three independent sex-matched, age-based groups: younger adults (YA), middle-aged adults (MA), and finally older adults (OA). To verify the ability of machine learning analysis to automatically classify handwriting samples according to age, all data were submitted to a convolutional neural network (CNN) algorithm. Furthermore, the performance of the artificial classifier was assessed in detail in all comparisons (i.e., sensitivity, specificity, positive and negative predictive values, and accuracy). Lastly, we also calculated the area under the receiver operating characteristic (ROC) curves to verify the optimal threshold as reflected by the associated criterion (Ass. Crit.) and Youden Index (YI).

## Materials and Methods

### Participants

We consecutively and randomly recruited 156 healthy subjects (61 males; mean age ± SD 49.6 ± 20.4 years, range 18–90 years) from the IRCCS Neuromed, Pozzilli (IS), Italy. All subjects were right-handed and native Italian speakers. We further divided participants into three age-independent subgroups to conform with previous demographic definitions of younger adults (YA) (18–35 years), middle-aged adults (MA) (36–55 years), and older adults (OA) (>56 years) (Petry, 2002). Accordingly, we examined 51 YA (23 males; 25.7 ± 3.2 years, range 18–32 years), 40 MA (17 males; 48.9 ± 5.9 years, range 37–57 years), and finally 63 OA (21 males; 71.3 ± 6.6 years, range 62–90 years). Anthropometric features including weight, height, and body mass index (BMI) were collected. The cognitive functions of all participants were assessed through the Mini-Mental State Examination (MMSE) (Folstein et al., 1975). None of the participants manifested cognitive or mood disorders, and none reported osteoarticular disorders or visual deficits significantly affecting handwriting. Also, no subjects were taking any drug affecting the central nervous system. The demographic (i.e., age and gender), anthropometric (i.e., weight, height, and BMI) and clinical (i.e., MMSE) features of the participants are reported in Table 1. All participants gave written informed consent, and the study was approved by the institutional ethics committee.

**TABLE 1:** Demographic and anthropometric features of participants at the handwriting task.

	Total number	Age (years)	Age range	Weight (Kg)	Height (cm)	BMI	MMSE
Participants	156	49.6 ± 20.4	18–90	69.5 ± 13.9	165.9 ± 8.9	25.3 ± 4.7	29.3 ± 1.1
YA	51	25.7 ± 3.2	18–32	61.4 ± 9.1	167.9 ± 7.8	21.7 ± 2.2	29.8 ± 1.0
MA	40	48.9 ± 5.9	37–57	73.8 ± 15.3	168.5 ± 8.2	25.9 ± 4.6	29.6 ± 0.8
OA	63	71.3 ± 6.6	62–90	70.3 ± 13.3	163.2 ± 9.2	26.4 ± 4.8	28.9 ± 1.3

*YA, younger adults; MA, middle-aged adults; OA, older adults; BMI, body mass index; MMSE, Mini-Mental State Examination. Results are expressed as average ± standard deviation (SD).*

### Handwriting Task

**FIGURE 1:**
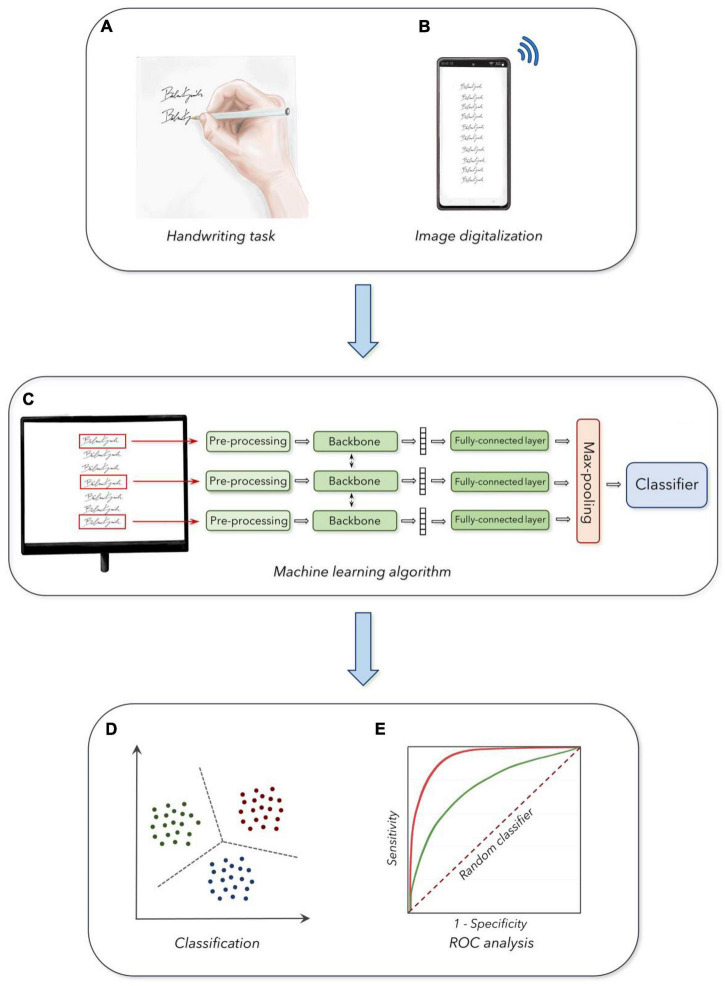
Experimental design: **(A)** Acquisition of handwriting samples. **(B)** Digitalization and collection of the handwriting task. **(C)** Machine learning analysis of handwriting samples. **(D)** Output of the classifier in the three age groups. **(E)** Receiver operating characteristic curves (ROC analysis) for the discrimination between the three groups (see section “Materials and Methods” for further details).

A written protocol with instructions on how to perform the handwriting task was sent to participants by the authors using the institutional email address. Also, following the enrollment, subjects received a preliminary supervised training trial to familiarize themselves with the experimental procedures. Then, participants were asked to perform the handwriting task while sitting on a chair, with their arms lying on a table, at home and in the morning. Concerning the handwriting task used in this study, participants were asked to write their own first and last names ten consecutive times on a paper sheet. We selected this specific task to exclude the influence of cultural factors and the contribution of higher cognitive abilities related to the symbolic aspects of handwriting potentially affecting the assessment of basic writing features. The first and last names were written starting from the upper and left sides of the document and then proceeding downward, in a single column, with a self-paced and comfortable speed. We preliminary provided participants with a fold of white A4 (210 mm × 297 mm) paper sheet (Fabriano, PG, Italy), and a couple of black ballpoint pen types (Bic, Clichy, France). After collecting the handwriting task three consecutive times, participants were asked to scan the signed paper sheet using the camera included in their smartphone (required resolution of at least 5 Megapixels). After scanning the handwriting samples, participants were asked to convert photos into portable document format (PDF) files using dedicated apps available for free download. Finally, participants completed the requested procedures by sending the PDF files to the authors’ institutional email server, and the files were stored anonymously on a dedicated Drive, encrypted, and password-protected (Figure 1).

### Handwriting Analysis

All handwriting samples were preliminarily assessed using a perceptive evaluation, to exclude corrupted files from subsequent analysis. Then, based on previous data reporting changes in stroke sizes in the elderly as a prominent age-related feature (Hilton, 1977; Dixon et al., 1993; Slavin et al., 1996; Walton, 1997; Contreras-Vidal et al., 2002; Teulings et al., 2002; Rodríguez-Aranda, 2003; Rosenblum and Werner, 2006; Burger and McCluskey, 2011; Caligiuri et al., 2014), we first automatically measured the average height of strokes in the handwriting samples in each participant. As a text detection method, we used the DBNet algorithm (i.e., ResNet-50 backbone), included in the docTR package, owing to its open-source availability and state-of-the-art performance for the task (Cao et al., 2020). The DBNet, was trained according to Cao et al. (2020) and was then applied to extract the precise bounding boxes corresponding to the handwriting samples^1^ (Cao et al., 2020). To limit the number of false-positive tests due to stylized strokes, we selected only bounding boxes with a height higher than 50 pixels (IC > 0.5) and relative to easily readable handwriting strokes. Finally, the DBNet algorithm calculated specific digital values as outcome measures.

The PDF files including the handwriting samples were further digitalized to fit with standard requirements for machine learning procedures, according to basic standards of artificial intelligence algorithms. A customized CNN model was built to perform classification in the age groups, following standard methodologies in the field of fine-tuning pre-trained models (Bengio et al., 2021). We classified pictures of single handwriting samples collected in the three age groups (i.e., YA, MA, and OA), according to standardized machine learning procedures, based on a fine-tuning of a pre-trained CNN with a randomly initialized fully connected layer at the end (Bengio et al., 2021). We developed a specialized *multi-view* CNN able to simultaneously process a set of *k* handwriting samples extracted from a single participant, to increase the robustness of the analysis (Figure 1). The size *k* of the input set was a hyper-parameter that was optimized with a dedicated grid search procedure. The architecture for further classification analysis considered the overall set as input. Accordingly, this architecture was composed of three blocks, a backbone which was used to independently process each sample in the set, a pooling block used to merge the outputs of the backbone and, finally, a classification block applied to provide a single prediction for the set (i.e., the age group of the handwriting sample). Then, after achieving a robust architecture, we organized the following steps consisting of the design of each component, the training procedure, and the hyper-parameters optimization procedures. First, the handwriting samples were resized to a single common dimension, to match the spatial dimension of the pre-trained backbone. Specifically, we resized all images to a 128 × 256-pixel proportion for all CNNs pre-trained on ImageNet (Bengio et al., 2021). To handle the handwriting samples characterized by images smaller than the optimal dimension, we applied white padding according to the respective size, matching the color of the underlying sheet of paper. We then converted the single out-of-scale image into a black and white negative image, thus obtaining large values for the corresponding output tensor. During training, we randomly selected subsets of *k* samples from the same PDF file as input to the network. We kept the amount of random rotation and translation to the inputs as lower as possible, to avoid image artifacts (Pang and Yang, 2016). All images were processed using a shared backbone, and we experimented with either (a) an AlexNet network trained from scratch, (b) a pre-trained ResNet-50 model, and (c) a customized Convolutional Recurrent Neural Network (CRNN), which was pre-trained on a task of optical character recognition (Wang et al., 2019). We included the choice of backbone as a second hyper-parameter to optimize the classifying procedure. We performed average pooling on the outputs of the backbone (across the spatial dimensions for the CNNs, and the temporal dimension for the CRNN), to associate each image in the input set with a fixed-dimensional embedding vector (i.e., size 1024 for the pre-trained ResNet-50 model). We applied a small fully connected layer with 100 hidden units and Gaussian Error Linear Units activation function (Hendrycks and Gimpel, 2016) on each embedding vector, and then we performed a max-pooling for all embeddings corresponding to the same input set, to obtain a fixed-dimensional vector for the entire set (Figure 1). The overall design was built to avoid the architecture being affected by the permutations of the samples in input (i.e., the output of the network does not change whether we shuffled the samples inside a set, and it could also potentially work for variable-sized sets). The final embedding vector was passed to a fully connected layer with three outputs (corresponding to the three age groups), trained with either a standard cross-entropy loss function or a weighted kappa loss function exploiting the fact that the three classes were naturally ordered (de la Torre et al., 2018; Figure 1).

To optimize the architecture, we randomly divided the dataset into three subsets with 80% of the subjects for training, 10% for validation, and 10% for test. We performed a grid search on the size of the input set, the choice of the backbone, and the selection of the loss function using the validation set, with the macro-averaged AUC score as a metric. The best performing architecture after the grid search, with a validation AUC of 81%, exploited the ResNet-50 model as the backbone, a set of size 3, and the cross-entropy loss, and we used these hyper-parameters in the following. All results shown in the experimental section were computed on the test set, by training over 50 different initializations and using the concatenation of the previously defined training and validation sets as training data. The final hyper-parameters of the model used in the experimental section were the following: the size of the crops (128; 256; RGB), number of views (Valdovinos et al., 2022), backbone model (ResNet-50—pre-trained), loss function (cross-entropy—multi-class). For the implementation, we used TensorFlow for the design of the model, with the pre-trained ResNet-50 weights taken from the official Google implementation in TensorFlow Hub, while the pre-trained CRNN weights were taken from the Keras-ocr repository.

### Statistical Analysis

The normal distribution of demographic and anthropometric features of subjects included in the YA, MA, and OA groups was assessed using the Kolmogorov-Smirnov test. The Chi-Square Test was used to compare the frequency of males and females in the three groups. The Mann-Whitney *U*-test was used to compare demographic and anthropometric parameters, as well as clinical scores (i.e., MMSE) in YA, MA, and OA. The Mann-Whitney *U*-test was also used to compare the stroke dimensions (i.e., heights) in participants from YA, MA, and OA groups. ROC analyses were calculated to identify the optimal cut-off values to discriminate between YA and MA, YA, and OA, and finally MA and OA, according to standardized procedures. For each ROC curve, we analyzed the Youden Index and its associated criterion (i.e., optimal threshold), as well as the Sensitivity (Se.), Specificity (Sp.), Positive Predictive Value (PPV), Negative Predictive Value (NPV), Accuracy (Acc.), Area Under the Curve (AUC), standard error (SE). Also, we compared AUCs of independent ROC curves to verify possible differences in statistical analysis, according to standardized procedures (Figure 1).

A *p*-value < 0.05 was considered statistically significant. Statistical analyses were conducted using STATA v17.0 (StataCorp LLC, United States).

## Results

Demographic and anthropometric variables were normally distributed among participants. The Chi-square test showed a balanced distribution of female and male participants within the three subgroups (chi-square: 1.48, *p* = 0.48). The Mann-Whitney *U*-test showed decreased weight and BMI in YA compared with MA (*p* > 0.05) and OA (*p* > 0.05), as well as decreased height in OA compared with YA (*p* > 0.05) and MA (*p* > 0.05).

We discarded less than 5% of all samples in case of a poor-quality acquisition of handwriting as evaluated through our preliminary perceptual analysis, and accordingly, these participants were asked to repeat the acquisition of new handwriting samples.

**FIGURE 2:**
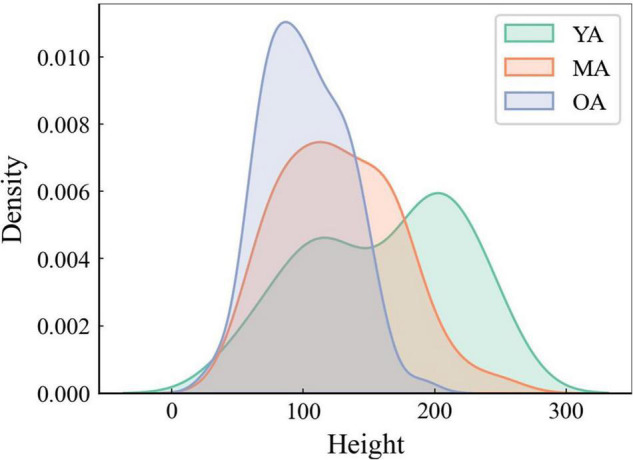
The average height of strokes analysis through DBNet algorithm. Note that the average height of strokes is smaller in OA than in MA and YA.

When comparing the average stroke dimensions (i.e., heights) achieved by using the DBNet software to the handwriting samples collected from YA, MA, and OA, the Mann-Whitney *U*-test showed smaller stroke heights in OA than YA and MA, as well as in MA than in YA (*p* < 0.01 for all comparisons) (Figure 2).

**FIGURE 3:**
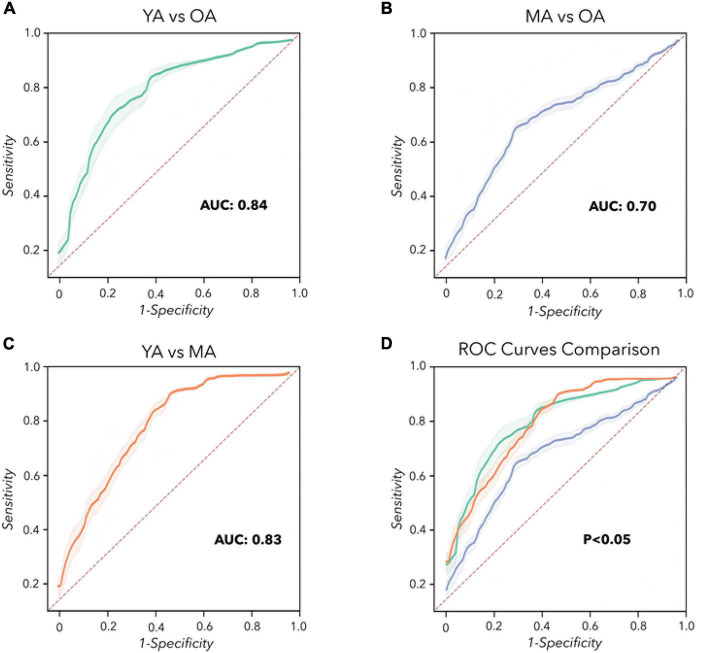
Convolutional Neural Network analysis. Receiver operating characteristic (ROC) curves were calculated to differentiate YA, MA, and OA. **(A)** YA vs. OA (green line). **(B)** MA vs. OA (blue line). **(C)** YA vs. MA (orange line). **(D)** Comparison of the ROC curves. The dashed red line represents the performance of a random classifier.

Concerning machine learning analysis, the CNN artificial classifier discriminated between YA and OA with high significant performance. The ROC curve analyses identified an optimal threshold value of 0.60 (associated criterion), when including 114 instances (Y.I. = 0.52). Using this cut-off value, the performance of our test was sensitivity = 82%, specificity = 80%, PPV = 78%, NPV = 79%, Acc. = 77%, and AUC = 0.840 (Figure 3A and Table 2).

**TABLE 2:** Performance of the CNN algorithm in classifying handwriting samples collected from the whole group of healthy participants.

Comparisons	Instances	Associated criterion	Youden Index	Se (%)	Sp (%)	PPV (%)	NPV (%)	Acc. (%)	AUC
YA vs. OA	114	0.60	0.52	82	70	78	79	77	0.840
MA vs. OA	103	0.46	0.40	84	56	78	73	74	0.700
YA vs. MA	91	0.59	0.63	75	82	79	83	79	0.830

*The performance of the CNN classifier was achieved for the comparisons between handwriting samples collected from three separate subgroups: (1) YA vs. OA; (2) MA vs. OA; (3) YA vs. MA. Instances refer to the number of subjects considered in each comparison (see section “Materials and Methods” for further details). YA, younger adults; MA, middle-aged adults; OA, older adults; Se, sensitivity; Sp, specificity; PPV, positive predictive value; NPV, negative predictive value; Acc, accuracy; AUC, area under the curve.*

The differentiation between MA and OA achieved by the CNN algorithm also disclosed significant performances. The ROC curve analyses identified an optimal threshold value of 0.46 (associated criterion), when including 103 instances (Y.I. = 0.40). Using this cut-off value, the performance of our test was sensitivity = 84%, specificity = 56%, PPV = 78%, NPV = 73%, Acc. = 74%, and AUC = 0.700 (Figure 3B and Table 2).

When discriminating YA and MA, the artificial classifier achieved a significant performance of the test. ROC curve analyses identified an optimal threshold value of 0.59 (associated criterion) when including 91 instances (Y.I. = 0.63). Using this cut-off value, the performance of our test was sensitivity = 75%, specificity = 82%, PPV = 79%, NPV = 83%, Acc. = 79%, and AUC = 0.830 (Figure 3C and Table 2).

When comparing the two independent ROC curves relative to the analysis of YA vs. MA and YA vs. OA, we obtained similar results: the difference between AUCs = –0.02, *z* = –0.205, *SE* = 0.098, *p* = 0.84 (Table 3). Also, when comparing the two ROC curves relative to the classification between YA vs. MA and MA vs. OA, the statistical analysis showed overlapping results: the difference between AUCs = 0.10, *z* = 0.726, *SE* = 0.138, *p* = 0.47 (Table 3). Finally, when discriminating between the two ROC curves relative to YA vs. OA and MA vs. OA, we demonstrated similar results: the difference between AUCs = 0.12, *z* = 0.822, *SE* = 0.146, *p* = 0.41 (Table 3 and Figure 3D).

**TABLE 3:** Comparisons of independent ROC curves.

Comparisons	Instances	AUC difference	Standard error	*z*-statistic	*P*-value
YA vs. MA – YA vs. OA	205	–0.02	0.098	–0.205	0.84
YA vs. MA – MA vs. OA	194	0.10	0.138	0.726	0.47
YA vs. OA – MA vs. OA	217	0.12	0.146	0.822	0.41

*Comparisons of the three independent ROC curves (i.e., YA vs. MA; YA vs. OA and MA vs. OA) were designed during the classification of handwriting samples collected from participants. Instances refer to the sum of the number of subjects considered in each paired comparison (see section “Materials and Methods” for further details). YA, younger adults; MA, middle-aged adults; OA, older adults; z-statistic, statistic output of the classifier; AUC, area under the curve.*

## Discussion

In the present study in a large cohort of healthy subjects, we demonstrated that physiologic aging deteriorates handwriting abilities. Specifically, we report, for the first time, that an advanced analysis based on machine learning algorithms, applied to a simple handwriting task collected in a real-life setting, objectively and automatically discriminated three independent sex-matched, age-based groups: YA, MA, and finally OA. The accuracy of the ROC curves here obtained pointed to a significant worsening of handwriting abilities led by physiological aging. Also, our study indicates that an ecologic machine learning-based analysis of handwriting in healthy subjects constitutes a reliable telemedicine approach, potentially useful for the future and remote recognition of neurological disorders.

### Handwriting and Physiological Aging

Our first analysis demonstrated significant smaller stroke sizes in OA than in YA and MA samples, suggesting an age-related progressive decline of handwriting abilities in healthy subjects. Our findings fully agree with previous observations reporting decreased sizes along with reduced velocities and pressures of strokes during handwriting in elderly people (Hilton, 1977; Dixon et al., 1993; Slavin et al., 1996; Walton, 1997; Contreras-Vidal et al., 2002; Teulings et al., 2002; Rodríguez-Aranda, 2003; Kim et al., 2005; Rosenblum and Werner, 2006; Burger and McCluskey, 2011; Plamondon et al., 2013; Caligiuri et al., 2014; Zham et al., 2019; Kanno et al., 2020). By contrast, some reports suggested increased rather than decreased stroke size in elderly subjects (Rosenblum et al., 2013) raising the possibility of relevant heterogeneity in previous methodologies and experimental approaches. Abnormal stroke sizes observed in the elderly during handwriting would point to the effect of physiological aging on brain networks contributing to this high-level cognitive function (Purcell et al., 2011; Planton et al., 2013; Bartoň et al., 2020). Clinical, neuropsychological and neuroimaging studies have demonstrated that handwriting relies on the widespread synergic activity of several brain regions, the *writing network*. More in detail, the angular gyrus (Roeltgen and Heilman, 1984) and the precentral gyrus (Rapcsak et al., 1988) contribute to lexical processes of handwriting, the left perisylvian regions play a role in phonological aspects (Roeltgen and Heilman, 1984; Rapcsak et al., 1988, 2009; Alexander et al., 1992b), whereas the left superior parietal or premotor regions are responsible for handwriting execution (Auerbach and Alexander, 1981; Anderson et al., 1990; Alexander et al., 1992a). Indeed, selective brain lesions in the angular gyrus/precentral gyrus/left perisylvian regions and in the left superior parietal/premotor regions are known to lead to dysorthographias (i.e., lexical or phonological components) (Roeltgen and Heilman, 1984; Rapcsak et al., 1988, 2009; Alexander et al., 1992b) and apraxic agraphia (i.e., grapheme tracing) (Auerbach and Alexander, 1981; Anderson et al., 1990; Alexander et al., 1992a), respectively. Lastly, the *writing network* also reflects the activation of subcortical structures such as the basal ganglia (i.e., striatum) and the anterior cerebellum (Kim et al., 2005; Purcell et al., 2011; Planton et al., 2013, 2017; Letanneux et al., 2014; Zham et al., 2019; Bartoň et al., 2020; Kanno et al., 2020). In the elderly, the age-related progressive reduction in stroke sizes during handwriting would therefore reflect structural or functional changes in cortico-subcortical components of the *writing network* (Purcell et al., 2011; Planton et al., 2013; Bartoň et al., 2020). Several previous reports have demonstrated an overall reduction of default subnetwork connectivity in elderly subjects when performing experimental paradigms assessing executive functions, such as handwriting (Planton et al., 2017; Schulz et al., 2022). This would reflect several age-related biological factors including progressive white matter involvement in the frontal lobe attributable to a small vessel disease (de Leeuw et al., 2001; Kim and Melhem, 2008; Walhovd et al., 2011; Hirsiger et al., 2017; Zhuang et al., 2018). A further consideration concerns the possible link between the age-related progressive reduction in stroke sizes and the parkinsonian micrographia. Previous observations have demonstrated that micrographia in PD is related to the severity of bradykinesia (Wu et al., 2016; Wyss-Coray, 2016; Schott, 2017; Canevelli and Marsili, 2022), thus reflecting dopaminergic striatal denervation (Hilton, 1977; Dixon et al., 1993; Slavin et al., 1996; Walton, 1997; Contreras-Vidal et al., 2002; Teulings et al., 2002; Rodríguez-Aranda, 2003; Rosenblum and Werner, 2006; Burger and McCluskey, 2011; Plamondon et al., 2013; Rosenblum et al., 2013; Caligiuri et al., 2014; Wu et al., 2016; Kanno et al., 2020). Accordingly, a further hypothesis would attribute the age-related reduction in stroke sizes observed in healthy elderly to abnormal activity in cortico-subcortical components of the *writing network* which at least in part overlap with those responsible for micrographia in PD (Kim et al., 2005; Rosenblum et al., 2013; Wu et al., 2016; Zham et al., 2019; Kanno et al., 2020). Future studies will disclose possible similarities between age-related reduction of stroke sizes and parkinsonian “consistent” (i.e., overall small handwriting) and “progressive” micrographia (i.e., serial reduction in handwriting size) (Kim et al., 2005; Rosenblum et al., 2013; Wu et al., 2016; Zham et al., 2019; Kanno et al., 2020). Despite PD, converging clinical, neuropsychological and neuroimaging evidence point to abnormal handwriting abilities also in patients with other neurodegenerative disorders including AD (Forbes et al., 2004; Delazer et al., 2021). Patients with AD may manifest agraphia associated with a reduction in stroke sizes (Delazer et al., 2021). Overall, given that micrographia constitutes a reliable writing feature in PD (Letanneux et al., 2014) and possibly characterizes also other neurodegenerative diseases such as AD (Delazer et al., 2021), we speculate that future handwriting analysis would help to assess the phenoconversion from physiologic aging into neurodegenerative disorders (Letanneux et al., 2014).

Relevant new findings also came from our machine learning analysis which allowed us to achieve high accuracy obtained when discriminating handwriting samples collected from YA and OA. This finding, which has been achieved objectively and automatically, strongly supports the hypothesis of a relevant detrimental effect of human aging on handwriting samples. Further relevant information on age-related changes in handwriting came from the high accuracy obtained when discriminating between handwriting samples from MA and OA. Given that our cohort of MA aged from 37 to 57 years, whereas the group of OA ranged from 62 to 90 years, our results would raise the intriguing hypothesis that handwriting prominently declines at the age of about 60 years. These findings agree with a previous study based on standard handwriting analysis which has shown a consistent deterioration of handwriting skills starting from 60 years of age (Verwey et al., 2011). This would reflect a relevant cognitive workload associated with a global reduced sensory processing performance leading to a prominent deterioration of handwriting skills in the elderly of similar age ranges (Engel-Yeger et al., 2012).

A rather unexpected finding of our study consists of the high accuracy achieved by machine learning when classifying between handwriting collected in YA and MA. Given that our cohort of YA ranged from 18 to 32 years, whereas MA aged from 37 to 57 years, our results would suggest early age-related changes in handwriting skills. The differences here observed when comparing handwriting between YA and MA would reflect several possible mechanisms. A first hypothesis concerns a putative early deterioration of brain networks responsible for complex motor tasks at about 35 years of age. Such a hypothesis would receive support from the observation that since the mid-twenties, complex motor, as well as cognitive functions progressively, decline (Berghuis et al., 2019; Stojan and Voelcker-Rehage, 2021). Given that brain networks regulating handwriting partially overlap with those controlling abstract thinking or complex motor planning, we believe that handwriting deteriorates in MA as a result of the relentless phenomenon of aging (Fraser et al., 2009). An alternative hypothesis would imply non-biological mechanisms, including social issues. Our group of YA consists of participants who belong to the so-called “millennial generation” and are possibly characterized by social- and cultural-driven changes in writing habits. The “millennial generation” indeed concerns teenagers and younger adults born since 1985 and refers to people that grew up in the internet age. These youngers largely use computers and other technological devices for writing their academic essays from the early school classes and for communicating on social media. Hence, instead of traditional writing, they prefer typing on a clipboard. Accordingly, it can be argued that this recently acquired social habit would impact significantly on handwriting skills in YA. Future studies could confirm our speculation on social-related changes in handwriting patterns in the “millennial generation” (Dressler et al., 2018).

A final consideration concerns the real-life setting of our experimental design. Indeed, we here report the first handwriting analysis based on machine learning to apply homemade recordings of handwriting samples. The high significance of the results achieved in such an ecologic scenario discloses plenty of future perspectives, including social distancing and disparities in the access to care (Chirra et al., 2019). Therefore, we suggest that future telemedicine studies on handwriting analysis in physiologic and pathologic aging should be based on the examination of their impact on daily activities, to improve daily performance and quality of life (Marsili et al., 2018; Marsili and Mahajan, 2022). Lastly, our study would also be useful to develop a methodology based on the automatic machine learning analysis of handwriting according to age. For instance, an automatic technique able to recognize age-related features of signatures would be applied by authorities to discriminate against authentic wills in heritage disputes or for dating documents and paintings.

We acknowledge that our study has potential limitations. Our sample size as well as the number of handwriting samples collected in healthy subjects would be considered relatively small. However, the accuracy achieved in the classification of handwriting in the three cohorts of healthy subjects was remarkable pointing to the reliability of our machine learning analysis. Also, given that we have not recorded handwriting samples serially in each participant, our study does not allow us to reach conclusions about the intrasubject variability in handwriting skills. Furthermore, we cannot fully exclude that the decreased weight and BMI observed in YA, and the decreased height observed in OA subjects would have contributed at least in part to our findings.

## Conclusion

In this study, by using an advanced handwriting analysis based on machine learning algorithms, we have objectively and automatically demonstrated that handwriting undergoes significant age-related changes. This would reflect age-related changes in the activity of cortico-subcortical components of the *writing network*. The high accuracy of our ROC curves analysis suggests that handwriting is a simple task that can be reliably used in a real-life setting for telemedicine purposes (Engel-Yeger et al., 2012; Canevelli et al., 2014; Camicioli et al., 2015; Marzinotto et al., 2016). Hence, we believe that our study provides the background for future applications in the field of telemedicine in patients with neurodegenerative disorders, including PD and AD. Also, we speculate that future investigations would benefit from concurrent recording and analysis of handwriting and other motor tasks (i.e., finger tapping) to objectively assess bradykinesia of the upper limbs in the elders as well as in patients with neurodegenerative disorders.

## Data Availability Statement

All clinical and instrumental data are stored offline and are available on reasonable request to the corresponding author.

## Ethics Statement

The studies involving human participants were reviewed and approved by the IRB of the IRCCS Neuromed Institute. Written informed consent to participate in this study was provided by the participants’ legal guardian/next of kin.

## Author Contributions

FA, SS, and AS: research project (conception). FA, SS, and LT: research project (organization). FA, AZ, VDO, LT, MP, and MF: research project (execution). FA, SS, LT, and AS: statistical analysis (design and execution). SS and AS: statistical analysis (review and critique). FA, LT and LM: manuscript preparation (writing of the first draft). SS and AS: manuscript preparation (review and critique). All authors contributed to the article and approved the submitted version.

## Conflict of Interest

LM has received honoraria from the International Association of Parkinsonism and Related Disorders (IAPRD) Society for social media and web support. The remaining authors declare that the research was conducted in the absence of any commercial or financial relationships that could be construed as a potential conflict of interest.

## Publisher’s Note

All claims expressed in this article are solely those of the authors and do not necessarily represent those of their affiliated organizations, or those of the publisher, the editors and the reviewers. Any product that may be evaluated in this article, or claim that may be made by its manufacturer, is not guaranteed or endorsed by the publisher.

## Footnotes

1
https://github.com/mindee/doctr
